# From prior knowledge to data-informed models: a review of Boolean network inference

**DOI:** 10.1093/bib/bbag499

**Published:** 2026-09-15

**Authors:** Pierre Klemmer, Ahmed Abdelmonem Hemedan, Reinhard Schneider, Marek Ostaszewski

**Affiliations:** Bioinformatics Core, Luxembourg Centre for Systems Biomedicine (LCSB), University of Luxembourg, 7 Avenue des Hauts-Fourneaux, Esch-sur-Alzette 4362, Luxembourg; Transversal Translational Medicine, Luxembourg Institute of Health (LIH), 1 A-B Rue Thomas Edison, Strassen 1445, Luxembourg; Bioinformatics Core, Luxembourg Centre for Systems Biomedicine (LCSB), University of Luxembourg, 7 Avenue des Hauts-Fourneaux, Esch-sur-Alzette 4362, Luxembourg; Bioinformatics Core, Luxembourg Centre for Systems Biomedicine (LCSB), University of Luxembourg, 7 Avenue des Hauts-Fourneaux, Esch-sur-Alzette 4362, Luxembourg

**Keywords:** systems biology, prior knowledge, boolean network inference, optimisation

## Abstract

Molecular mechanisms are highly complex and involve numerous components in non-linear interactions, imposing challenges in analysis and understanding. Networks provide intuitive representations of these systems, enabling visualisation of experimental data and the aggregation of prior knowledge. However, static networks cannot account for key temporal dynamics. Boolean networks address this limitation as discrete dynamical models representing temporal properties of biological regulation through binary variables connected by logical functions, offering scalable frameworks for hypothesis generation while avoiding parameterisation challenges of quantitative modelling approaches.

Construction of (Boolean) networks is a mostly manual, labour-intensive and bias-prone process, relying on the review of a large corpora of prior knowledge. This review surveys methods for Boolean network inference that integrate experimental data with or without prior knowledge to automatically construct context-specific, data-informed models. We begin by examining network curation methods from text mining to manual curation given their use as backbones of Boolean networks, then analyse several inference algorithms categorised as heuristic (MIBNI, ATEN, LogicGep, CANTATA, and CellNetOptimizer) and exact (BoNesis, caspo, Sketchbook, RE:IN, and Griffin) methods. For each method, we discuss computational representation, inputs, inference algorithms, and benchmarking.

We identify key considerations for method selection, including handling of uncertainty, choice of updating scheme, scalability, and data discretisation challenges. We advise that method selection be guided by the type and extent of available prior knowledge and experimental data, and modelling objectives.

## Introduction

Analysis of molecular mechanisms is challenging due to their complexity and scale; interactions between biomolecules are often non-linear and involve multiple feedback loops and dozens of participants. Such complex mechanisms can be represented as networks of interacting elements, providing an intuitive illustration and a systematic framework for aggregating knowledge from different sources [1, 2]. Networks are usually constructed through manual curation based on prior knowledge sources like literature or interaction databases [1–3] or inferred from data [4]. They can be used for visualising the structure of interacting molecules [5–7], and can support exploration of complex data [8, 9]. Computational analysis of network structure can identify globally important properties and elements, like betweenness centrality or hub elements, and fine-grained details [10, 11]. However, static networks are unable to represent changes over time, leading to important limitations in model accuracy.

Various modelling approaches have been proposed to analyse dynamic properties of molecular and cellular systems, including ordinary differential equations [12, 13] or statistical models [14]. Despite allowing for granular simulation and analysis of complex systems, they are specific to the formal representation of the model, the parameters used for its description, and the modelling software used to run it. Also, computational models face scalability challenges related to parameterisation and computation time; in fact, the parameters required to calibrate such models are frequently unknown, prohibiting accurate simulation [15–17].

Network models are a hybrid approach, where a network is used as the topological structure of a model, and its elements and interactions are parameterised to enable analysis of system dynamics, capturing key temporal properties of biological regulation [18, 19]. Examples of such approaches include Boolean networks [20, 21], Petri nets [22], and Bayesian network models [23]. Boolean networks represent biomolecular interactions as discrete dynamical systems where nodes represent genes, proteins, or cellular states as binary (on/off) variables updated via logical rules. In contrast to unstructured approaches as mentioned in the previous paragraph, network models are relatively simpler to parametrise as they are typically ignorant of precise kinetic and stoichiometric parameters. Nevertheless, they offer a plausible trade-off between static networks and quantitative modelling frameworks.

In this article, we focus on Boolean network (BN) models, which benefit from straightforward implementation given their independence from specific biochemical parameters at the cost of precision of prediction. Gene expression or protein abundance are abstracted to binary states, and BNs cannot predict precise timing of molecular events as they lack quantitative temporal parameters.

Despite these limitations, they scale well to produce rich representations of complex systems and are used as hypothesis-generating tools, evaluating the impact of perturbations like mutations or pharmacological interventions on the system as a whole [21, 24, 25]. Another advantage of BNs is that they can be constructed in a straightforward way, using existing networks like manually curated pathway diagrams [26, 27] or disease maps [21], or through a similar curation process as static networks.

De novo construction of a BN yields high-confidence representations, but is a slow and labour-intensive exercise, requiring the synthesis of a vast corpus of biomedical publications by domain experts [2, 28]. During this process, the scope of BNs can be limited by subjective interpretation of evidence, or gaps in expert knowledge or literature used in curation. By complementing prior knowledge sources with novel experimental data, network models can be inferred at greater speeds, scales, and specificity for more efficient analysis and prediction of molecular mechanisms [29, 30].

This review surveys available network inference approaches, focusing especially on Boolean network inference because of its simplicity, scalability, and utility. We begin by briefly describing the automatic and manual curation of static networks before concluding with a detailed analysis of heuristic and exact optimisation approaches for Boolean network inference. In contrast to other reviews of Boolean network inference [31, 32] providing applied benchmarks, we aim to give a comprehensive overview of the Boolean network inference process.

We emphasise the curation and integration of prior knowledge for inference and highlight considerations like interoperability and algorithm inputs to evaluate inference methods beyond the inference algorithm itself.

## Definitions

### Network

A *network*  $G=\left(V,E\right)$ is constituted of a set of *vertices*  *V* and a set of *edges*  *E*. *Directed* networks are commonly used to communicate the orientation of interactions, defined as $G=\left(V,E,\phi \right)$ where *ϕ* indicates a function mapping every edge to an ordered pair of vertices: the first vertex is referred to as *source*, or *regulator*, which affects the second vertex called *target*. They are graph-based models of biological systems: vertices, hereafter referred to as *nodes*, represent genes, proteins, metabolites, or other biomolecules connected by edges representing interactions or associations.

### Boolean network

*A Boolean network*  ${F}^{(n)}$ is a type of qualitative, discrete network model consisting of *n* Boolean variables ${Var}_n=\left\{{v}_1,\dots, {v}_n\right\}$ assuming a Boolean value $B=\left\{1,0\right\}$, representing nodes and their respective activity or presence. Network dynamics are regulated by a set of *n* Boolean update functions $\left\{{F}_1,\dots, {F}_n\right\}$, which specify how the state of each node depends on its regulators.

## Prior knowledge network curation

Networks are constructed through automatic and manual curation processes [33]. Automatic approaches infer network structure from experimental data or prior knowledge using statistical methods, enabling high-throughput network generation at the cost of specificity and reliability [4].

Manual approaches deliver refined networks [34] derived from literature and database searches or preexisting models, reviewed by biocurators and domain experts [1–3].

### Automatic curation

Advances in accessible [35], high-throughput experimental data and natural language processing (NLP) make data-based network inference increasingly feasible. NLP methods can parse significant amounts of scientific publications to automatically extract relevant knowledge.

**Text mining** Text mining extracts and converts knowledge from publications and databases to standardised, machine-readable representations. Named entity recognition is essential in automatically processing and evaluating text to extract relevant entities and relationships between them automatically, constituting a key role in both heuristic and statistical text mining systems [36].

Large-scale NLP systems like Reach [37] parse scientific publications to extract interactions using statistical evaluation, heuristically identifying the semantic meaning and structure of sentences. The Integrated Network and Dynamical Reasoning Assembler (INDRA) [38] combines multiple such NLP systems [37, 39–43] to identify biomolecules from input texts using context-inclusive ML models [44, 45], then determines their logical meaning using semantic and ontological lexicons. Language models have garnered significant attention for their versatility in parsing and identifying large amounts of text through statistical models, parsing text structure and identifying e.g. relationships between entities [46].

BioBERT [46] and PubMedBERT [47] are contextualised encoder-only word representation models based on the BERT [48] language model, pre-trained for biomedical text mining on PubMed abstracts and PMC full-text articles to achieve improved performance in named entity recognition, relation extraction, and question answering tasks.

Similarly, BioNER-LLaMA [49] is an extension of the decoder-only LLaMA models [50, 51], performing NER through instruction tuning based on disease [52], chemical-disease [53], and gene–gene product [54] datasets, enabling repurposing of the model from NER to relation extraction. While decoder-only models (e.g. BioBERT) outperform encoder-only (e.g. BioNER-LLaMA) models in NER, they impose longer inference times and demanding hardware [55].

Natural language processing offers an efficient approach to rapidly parse and interpret a large corpus of scientific literature. Extracted relationships are standardised to enable parallel processing using several NLP systems for high-fidelity representations. Relationships can include connectivity logic or kinetic parameters, providing important utility for the construction of dynamic models. However, it is difficult to evaluate contradicting or ambiguous statements; rather than being a technical obstacle, such irregularities are difficult even for researchers to address. Language inconsistencies, such as misspellings and complex sentence structure pose considerable challenges for text mining. Nevertheless, this approach is valuable for automated identification of potential building blocks for network models [38, 56, 57].

**Data-driven network curation** Networks can be generated purely from experimental data, establishing undirected links between biomolecules through correlation [58, 59], and directed links using Bayesian statistics [60], regression [61] or mutual information [62–64] approaches. Inference of directed networks is typically limited to small networks due to prohibitive computational cost [60, 61, 64, 65]. Prior knowledge and experimental data may be combined to predict directionality, such as in D’or [66], where a deep learning framework utilises known cause-effect pairs from perturbation experiments and network propagation to assign directionality in undirected protein–protein interaction networks. Alternatively, prior knowledge and data can be combined for networks visualising putative interactions between particularly relevant data points by querying interaction databases like STRING [67] for sets of differentially expressed genes identified from data analysis [11].

### Manual curation

Context-specific, intended use of a network dictates its size, context, and coverage [1–3].

In manual, knowledge-based approaches, curators may use manually curated pathway (e.g. Reactome [68], the Disease Maps Project [69]) and interaction (e.g. DisGeNET [70], STRING [67]) databases. Such resources house vast amounts of biomolecule interactions, and often include scores based on evidence type or quality.

Given the effort required in manual curation and the rapid publication of new findings, manually curated databases especially lag behind the most recent research and rarely contain networks suitable for specific research questions: the aggregate database Pathway Commons [71] is estimated to cover only ~1%–3% of the currently available biomedical literature [56]. Because of low coverage of mechanisms of interest, curators perform systematic literature searches to extract interactions from scientific publications based on reliable experimental methods, identified based on domain expertise and network scope [1, 28]. Manually curated knowledge offers a high degree of confidence in the represented information. Curators take biological and experimental context into account and typically consult with experts in the field to deliver quality knowledge representations.

Despite efforts in publishing guidelines [1–3, 28, 69] and standards [72] for manual curation, the process is invariably prone to bias since curators may interpret the same evidence differently, exacerbated by project-specific considerations, while the volume of biomedical information published every day makes it challenging to keep up with the latest knowledge [73].

## Introduction to Boolean network inference

Boolean networks (BNs) are logic models with a topological structure representing the relationship between discrete Boolean variables by sets of Boolean functions (BFs). In BNs, Boolean variables and functions are analogous to network nodes and sets of edges, where the state of a given node corresponds to biomolecule activity (e.g. on or off) as a consequence of incoming regulatory edges [20, 74].

The combination of multiple regulators by logical connectives AND, NOT, and OR (frequently denoted as ∧, ¬, ∨, respectively) introduces complex regulatory relationships between model components. Boolean networks are simulated using various updating schemes guiding signal propagation and state trajectories (i.e. the sequence of model states) [20, 74]. Synchronous updating schemes deterministically update the states of every node at each transition (change from one system state to the next), while asynchronous schemes update one random node state per transition [75]. Model dynamics can be used to determine attractors—states or sets of states that the model cannot escape from.

Attractors represent long-term system behaviour and can be understood as biological phenotypes when they correspond to specific configurations of molecular markers and remain stable across simulations.

Point attractors refer to single stable states, while cyclic attractors represent a repeating sequence of states. Attractor search in combination with perturbation analysis is a powerful tool to determine how a model behaves after a given intervention, i.e. whether the forced inhibition of a protein target results in e.g. cell death. While dependent on model structure and logic, point attractors are independent of updating scheme and are considered robust. In contrast, cyclic attractors’ existence is closely linked to the update scheme [76, 77], making them specific to the set of model parameters.

Boolean networks can be manually constructed or derived from curated interaction networks using tools like CaSQ [26], integrating rulesets to convert interaction networks built using CellDesigner [78, 79] to Boolean models.

To overcome limitations in curation and network specificity, Boolean models can be inferred directly from experimental data or through a combination of data and prior knowledge. This approach accelerates model construction and yields context-specific models with improved predictive accuracy with respect to held-out experimental observations. Integrating experimental data during inference is especially valuable because the model’s dynamics can be compared to observed data, providing insight into how closely the inferred network resembles the real biological system.

In general, Boolean network inference methods seek to determine the most valid network structure and behaviour by identifying the optimal configuration of nodes and interactions that best reflects the experimental data [80–82].

Inference is formulated as a search and optimisation problem, where for *n* components, the search space comprises ${2}^{2^n}$ candidate BFs. Optimisation approaches systematically explore or restrict this space to identify candidates best describing observations. To verify if candidates capture system dynamics, most methods integrate simulation during inference.

**Figure 1:**
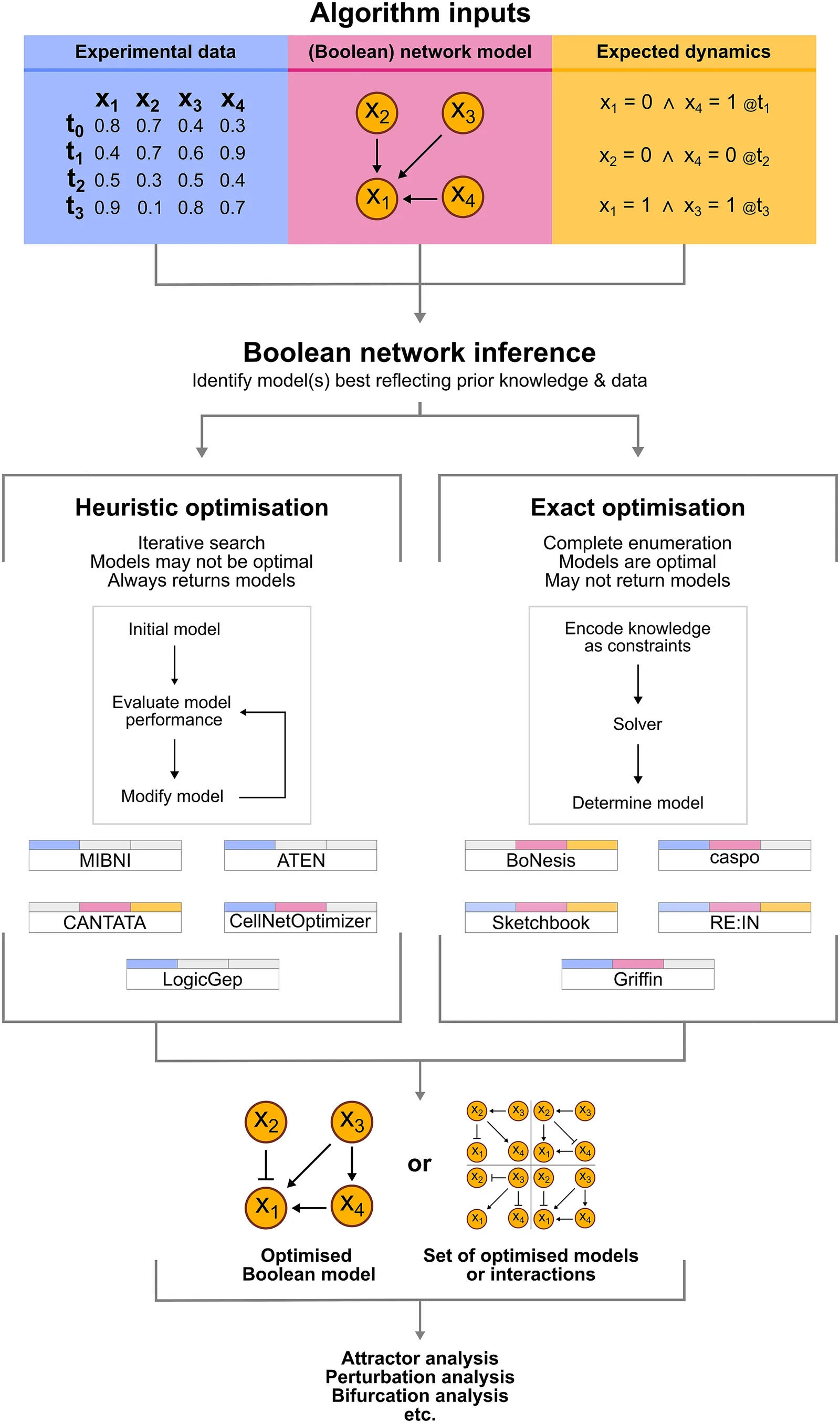
Boolean network inference workflow from experimental data (blue), network structure (pink), and expected dynamic behaviour (yellow). Coloured bars above algorithm names indicate expected inputs. Methods are grouped by optimisation strategy.

An overview of Boolean network inference approaches covered in this review can be found in Fig. 1. The rationale for the set of covered methods can be found in section S1 of the supplementary information. In brief, we consider heuristic optimisation methods like MIBNI [83], ATEN [84], LogicGep [85], CANTATA [81] and CellNetOptimizer [86, 87] as well as exact optimisation approaches including BoNesis [88, 89], caspo [80, 90, 91], Sketchbook [92, 93], RE:IN [94], and Griffin [95].

## Computational representation of models

The computational representation of BFs and models during inference (Table 1) influences performance by e.g. compressing redundancies in prior knowledge, increasing inference scale. The disjunctive and conjunctive normal forms specify how sets of literals (biomolecules) are connected by the OR and AND operators, respectively—e.g. (x_2_ ∧ x_3_) ∨ x_4,_ where (x_2_ ∧ x_3_) and x_4_ make up one clause respectively, separated by the OR operator. Symbolic trees are natural representations of BFs in genetic programming, while hypergraphs are commonly used to display networks. Binary decision diagrams compress isomorphisms and enable direct operation on this compressed form, resulting in considerable computational efficiency. RE:IN uses bit vectors for symbolic representation.

**Table 1:** Model representations used by the methods covered in this review along with visual examples.

**Representation type**	**Example**	**Used by**	**Advantage**	**Disadvantage**
Disjunctive normal form	${x}_1=\left({x}_2\wedge{x}_3\right)\vee{x}_4$	MIBNI, BoNesis	Standard way to encode Boolean functions, input for solvers	Impractical for large functions, redundancy
Conjunctive normal form	${x}_1=\left({x}_2\vee{x}_4\right)\wedge \left({x}_3\vee{x}_4\right)$	Griffin		
Symbolic tree	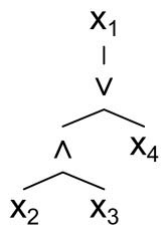	CANTATA	Easily interpretable, intuitive, computationally efficient	Redundancy, no unique representation
Directed hypergraph	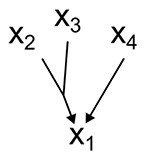	CellNetOptimizer, caspo	Common network representation for complex relationships, intuitive	Computationally expensive
Binary decision diagram	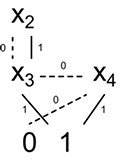	Sketchbook, Griffin	Compresses isomorphisms, computationally efficient	Unintuitive, less efficient for small functions
Bit vector	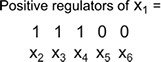	RE:IN	Computationally efficient, input for solvers	Unintuitive
K-expression	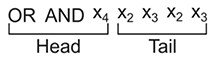	LogicGep	Computationally efficient, guarantees valid trees	No unique representation

Regulation graphs as introduced in the Griffin tool allow the explicit representation of known and suspected interactions with considerable detail (e.g. an interaction may not be required, but if it exists, it should be positive) in propositional logic sentences encompassing all networks consistent with the regulation graph and given biological constraints, which are then converted to a conjunctive normal form for SAT solving.

LogicGep represents candidate BFs as K-expressions, enabling efficient candidate modification and ensuring validity of corresponding symbolic trees.

## Inference algorithm configuration

Prior knowledge networks or existing Boolean models guide inference by detailing known or suspected interactions between model components. The use of data reinforces model inference; especially time-resolved datasets increase confidence that the inferred model accurately approximates underlying dynamics of the observed system [31]. Because different inference procedures have differing aims, many specific types and formats of prior knowledge and data are custom to individual methods; the types of input information are highlighted as coloured bars for each method in Fig. 1.

Experimental data used in Boolean network inference typically needs to be discretised to binary values for parametrisation and to evaluate the error between model prediction and experimental observations. Importantly, relative sparsity of temporal observations alongside inherent differences in gene expression magnitude imply that a simple global discretisation threshold (i.e. a value below/above which data points are set to 0/1) is not appropriate for biological data.

The multiscale binarisation approach [96] may be more suitable: time-resolved measurements of each gene expression are used to identify individual binarisation thresholds, discarding entries which are too noisy for confident discretisation. Regardless of approach, discretisation invariably removes biological variation by approximating relative biomolecule quantity to either 0 or 1. It should be noted that the chosen discretisation approach has significant impact on inference outcomes; a thorough comparison of discretisation approaches for Boolean modelling can be found in [31].

While most tools covered here expect data to already be binarised, others implement discretisation procedures; for instance, MIBNI and LogicGep infer Boolean networks solely based on time-series gene expression data, which is binarised using gene-wise k-means clustering. It should be noted that in contrast to the multiscale approach by Hopfensitz et al. [96], k-means clustering always returns two clusters of expression even if the expression data does not have a bimodal distribution.

Moving to methods lacking internal binarisation, the BoNesis software utilises existing influence graphs or partially specified Boolean networks to represent prior structural knowledge in the AEON format [97] together with expected dynamic behaviour and state reachability under normal or perturbed circumstances derived from prior knowledge or experimental data.

CANTATA makes use of the BoolNet [98] format to specify the prior knowledge network and a separate rule file containing expected dynamic behaviour and attractors of the model based on experimental observations as a list of active and inactive model components.

ATEN can perform inference solely based on binarised time series data, though prior knowledge may be incorporated by specifying e.g. the expected number of regulators for a given target gene and fixing certain regulators to always be included in solutions.

CellNetOptimizer and caspo employ the MIDAS format to represent experimental conditions and observations [99] and use a multi-value normalisation scheme for experimental data involving instrument sensitivity, detection thresholds, and well-characterised observations in the context of the given perturbation experiment. Based on the MIDAS file, CellNetOptimizer and caspo reduce the prior knowledge network, represented in the SIF format, by maximally removing redundant elements and their interactions [100], aiming at reducing the search space of possible Boolean models.

Moreover, Boolean network sketches [92], integrated in the Sketchbook [93] application, specify prior knowledge networks using a custom JSON format, SBML-qual [101, 102], or the AEON format to integrate definite and potential influences between model components, dynamics, and a comma-separated text file to represent binarised experimental data.

In Griffin, network structure and behaviour are specified using a Griffin-specific syntax to specify lists of components, known or putative interactions, known or putative attractors, and parameters for the inference.

RE:IN uses one file specifying the updating scheme, putative and known interactions, time-series observations, and stable states in a proprietary format, assuming that continuous variables have already been discretised.

Though common formats like SIF or SBML-qual are used by e.g. caspo and Sketchbook, most of the tools covered in this review implement their own standards and formats (Table 2), reducing interoperability and imposing challenges in applying several methods to the same inference problem. Similar issues can be observed for tools intended for Boolean model analysis and simulation [20], though efforts like the CoLoMoTo [103] and the COMBINE [72] initiatives aim to impose standards for interoperability and reusability of models.

**Table 2:** Formats for prior knowledge and data, as well as programmatic environments for each method covered in this review (N/A = not applicable). ^*^All algorithms are publicly available; MIBNI is available upon request to the authors.

**Method**	**Prior knowledge format**	**Data format**	**Environment**
CANTATA	BoolNet	Own format (Boolean expressions of expected attractors)	C++ (CLI)
CellNetOptimizer	SIF	MIDAS format	MATLAB, R, Python, Cytoscape
caspo	SIF	MIDAS format	Python
Sketchbook	Custom JSON, AEON, SBML-qual	Own format (Tabular data of expected attractors)	Rust (Standalone app)
BoNesis	AEON, BoolNET, SIF, own format	Own format	Python
MIBNI^*^	N/A	Own format	Java (CLI)
ATEN	N/A	Own format	R
RE:IN	Own format	Own format	F#
Griffin	Own format	Own format	Java (CLI or API)
LogicGep	N/A	Own format	Python

## Inference algorithms

NP-hard problems like Boolean network inference [104] may be addressed by numerous optimisation procedures using varying types of prior knowledge and experimental data, aiming to efficiently reduce and explore the search space of possible Boolean models (see ‘Introduction to Boolean Network Inference’).

The following section reviews optimisation approaches ranging from heuristic to exact methods, outlining their key considerations and summarising them in Table 3.

**Table 3:** Overview of discussed inference methods (O1 = Distance between model trajectory and data, O2 = Model size, O3 = Structural similarity to prior knowledge, O4 = Sensitivity to noise, N/A = not applicable).

**Method**	**Optimisation approach**	**Algorithm**	**Objective(s) to minimise**	**Updating scheme used for inference**
MIBNI	Heuristic	Optimisation using mutual information	O1	Synchronous
ATEN	Heuristic	Ensemble learning, simulated annealing	O1	Synchronous
LogicGep	Heuristic	Gene expression programming	O1, O2	Synchronous
CANTATA	Heuristic	Genetic programming	O1, O2, O3, O4	Synchronous
CellNetOptimizer	Heuristic	Genetic algorithm	O1, O2	Signal propagation
BoNesis	Exact	Answer set programming	N/A	Most permissive Boolean network semantics
caspo	Exact	Answer set programming	O1, O2	Signal propagation
Sketchbook	Exact	Binary decision diagram solving	N/A	Asynchronous
RE:IN	Exact	Satisfiability modulo theory solving	N/A	Synchronous, asynchronous
Griffin	Exact	Boolean satisfiability problem solving	N/A	Synchronous

### Heuristic optimisation approaches

Heuristic search strategies aim to provide near-optimal solutions by making incremental, stochastic improvements to candidate solutions guided by some objective measure.

They are used for problems with large search spaces that prohibit finding globally optimal solutions with acceptable computational cost [105].

**Heuristic search** Data-driven inference methods require specification of the maximum number of regulators for a given target to avoid combinatorial explosion of candidate BFs.

MIBNI infers Boolean models from time series gene expression data, employing a mutual information feature selection approach using a synchronous updating scheme to first identify regulators maximising pairwise mutual information with targets, then permute this set of regulators and their signs (positive or negative influence on the target) to increase model dynamics consistency.

The ATEN algorithm uses symbolic tree ensemble learning to infer Boolean models using a synchronous updating scheme by bootstrapping binarised time series data to construct an ensemble of candidate BFs for each target node. Simulated annealing, in which clauses and regulators may be added, swapped, or deleted, is used to reach near-optimal consistency with the data. Then, the importance of each clause is assessed by adding or removing it from the candidate BFs to observe whether prediction accuracy improves. Finally, a BF of minimal size is inferred from the remaining clauses before they are combined into a Boolean network.

In LogicGep, time-resolved bulk or pseudotime-resolved single cell transcriptomic data is used to infer individual BFs which are finally combined into a Boolean network model. Putative regulators are identified using random forest and XGBoost model fusion on binarised data before integrating them with gene expression programming to randomly modify (crossover and mutation) and select candidate BFs. Top candidates are discriminated using neural networks, evaluating which candidate regulator sets achieve the best performance in predicting target expression based on the original continuous data.

The CANTATA algorithm integrates Boolean models with a series of expected system states to infer optimised models under the synchronous updating scheme. It uses genetic programming with random modifications of candidate BFs, like insertion of a new logic operator or biomolecule node, deletion of a subtree, operator inversion, or negation of a subtree, as well as injecting modified versions of the initial model at each generation to explore candidate solutions.

CellNetOptimizer infers Boolean models of signal transduction for short-term dynamics under a signal propagation scheme [106] from a reduced Boolean model and perturbation experiment data (see ‘Inference Algorithm Configuration’) using a genetic algorithm, applying crossover and mutation with stochastic universal sampling to select candidate networks while avoiding premature convergence. It incorporates an elitism scheme, selecting the top individuals for the next generation without changes. At the final step, edges not affecting the fitness are removed.

In contrast to CANTATA, CellNetOptimizer and LogicGep permit a more liberal exploration of the search space given their ability to apply crossover to candidate solutions.

**Objective functions** Objective functions, also called fitness functions, dictate how candidate models are evaluated, guiding heuristic search approaches. In Boolean network inference, objective functions usually involve minimising the error between model prediction and experimental data, as well as minimising model size.

In MIBNI and ATEN, regulator candidates are assessed by comparing the trajectories (see ‘Introduction to Boolean Network Inference’) of the inferred candidate network model with the target, represented by binarised experimental data. Candidates are swapped using SWAP and recursive feature reconstruction and elimination routines until a minimum difference between trajectories is found.

In lexicographic optimisation, objectives are ordered by priority, and the first objective dominates all others. A candidate with an excellent first objective but a poor second objective is therefore preferred over one with moderate performance on both, eliminating the need for context-dependent weighting of objectives.

CANTATA applies a lexicographic fitness function to the entire model based on similarity between predicted model trajectories and data, size and structural similarity of the inferred functions to the input prior knowledge network, and ability to recover similar successor states from marginally different starting states.

LogicGep also uses a lexicographic fitness function to balance between predicted model trajectory and data, and candidate function size.

CellNetOptimizer utilises a fitness function applied to the entire model, incorporating model dynamics evaluated using mean squared error between model prediction and experimental observation, and a weighted size penalty.

### Exact optimisation approaches

While heuristic approaches are typically simple to implement and have lower hardware requirements, they cannot guarantee globally optimal solutions [105]. In exact approaches, candidates are identified by adherence to a set of criteria, often representing expected steady states or dynamic behaviour. More specific constraints lead to a smaller set of accepted solutions, making exhaustive search feasible and allowing for extensive integration of prior knowledge for inference.

In the BoNesis software, answer set programming (ASP) is combined with a model-checking approach based on most permissive Boolean networks where nodes can take intermediate states [107, 108], integrating prior knowledge with time-series data to reduce the search space by checking whether candidates can reach specific states with the clingo solver [109], omitting the need to explicitly simulate candidates.

The caspo approach uses ASP to learn Boolean models from prior knowledge networks and perturbation data using the clasp solver [110] and a signal propagation scheme [106]. Deliberate lack of feedback loops implies that inferred models are incapable of complex behaviour or structure. Caspo uses a lexicographic optimisation approach to further reduce the number of identified solutions, with minimising (i) the residual sum of squares between experimental observations and Boolean model predictions, and (ii) the length of the given BF as objectives.

Sketchbook identifies all candidates consistent with existing networks and potentially complex BFs under the asynchronous updating scheme by default, though synchronous and most permissive semantics could also be used. Inference is reinforced by checking for dynamic properties through the implementation of hybrid computation tree logic to describe expected attractors or state transitions. These reflect prior knowledge about the system and its behaviour derived from partial prior knowledge networks [108] or experimental data.

The RE:IN method [94] integrates definite and putative network components and BFs, known regulation, state transitions, and experimental insights under synchronous and asynchronous updating schemes, using satisfiability modulo theories together with bit vectors and the Z3 solver [111] to automatically reason on the existence and structure of consistent Boolean networks which may be further constrained to return e.g. the smallest consistent network.

The Griffin tool leverages a SAT solver [112] and binary decision diagrams for symbolic representation and optimisation of Boolean networks using the synchronous updating scheme. It combines prior knowledge networks and biological constraints to represent known and putative interactions and attractors of both wild-type and mutant organism states. Clause learning and blocking approaches are used to avoid the need to explicitly check for e.g. states the model should not be able to reach. A query splitting option in which all optional regulations are first removed, then gradually added back is also available for increased inference speed. Importantly, Griffin does not integrate time-series data for inference.

## Evaluation and benchmarking

Benchmarks deliver key insights into the accuracy of inference methods and could provide opportunities to compare different algorithms. Importantly, it should be noted that systematic inter-algorithm benchmarks are lacking, therefore making the comparison of different inference algorithms challenging. In common intra-algorithm reconstruction benchmarks, used for MIBNI, ATEN, LogicGep, CANTATA, CellNetOptimizer, and caspo, a reference Boolean model is used to generate synthetic data before either undergoing random topological changes or introducing noise to the synthetic data. The data and networks are then used as inputs for inference algorithms, aiming to reconstruct the reference Boolean model.

Approaches like BoNesis, Sketchbook, or RE:IN benchmark reduction in search space and computational cost gained through the integration of prior knowledge and expected dynamic behaviour as model constraints, assessing how different integration of constraints affects inference accuracy. However, the reported reduction in candidate Boolean models is closely linked to the different assumptions, constraints, and models chosen for their respective benchmarks.

Griffin is tested on several queries with differing aims, generally looking to infer Boolean models producing known dynamic behaviour, as well as testing hypothetical interactions and dynamic behaviour for consistency.

Table S1 in the supplementary information summarises, for each method, the types of benchmarks performed in the original publication along with details of the tested model/prior knowledge network and data provenance.

Pušnik et al. performed benchmarking of two data-only methods covered in this review (MIBNI and ATEN) alongside others (Best-Fit [113], GABNI [114], and REVEAL [115]) on the same E.coli model with associated data [116] for structural and dynamic accuracy [32].

As expected, the dynamics accuracy of inferred networks decreases with growing model size, though all methods performed similarly well. The authors note that structural validation of inferred models is a poor measure for performance; solely considering the topology of a Boolean network model entirely discards the dynamics these models benefit from. As such, it is important to properly define the expected dynamic properties (e.g. transitions, attractors, etc.) under a given set of parameters including updating scheme before benchmarking.

Furthermore, structural accuracy metrics suffer because of biological degeneracy, i.e. that different regulatory structures can result in the same dynamic behaviour. Given that only data-driven methods are benchmarked, the authors point out the utility of prior knowledge in increasing inference accuracy.

They conclude that none of the methods clearly outranks the others; only that REVEAL is overly sensitive to noisy data, and that methods overestimating the number of regulators (e.g. GABNI and MIBNI) have higher accuracy and recall but worse structural accuracy.

Jiang et al. compare caspo, CellNetOptimizer (using genetic algorithm and integer linear programming) and MEIGO [117] using a reconstruction benchmark based on a small model and a dataset [118], a larger model dataset from the DREAM3 challenge [119], and a T-cell model [106] with synthetic data [120].

The authors conclude that CellNetOptimizer provides strong topological performance at the cost of computation time while caspo appears to struggle in the reconstruction of noisy networks, though it remains fast across noise levels and model sizes. Similarly to Pušnik et al., the authors find that inference accuracy decreases with network size and structural noise and that common performance metrics like precision, accuracy, Hamming distance, or mean squared error are inconclusive for comparing inference methods.

Benchmarking of Boolean network inference methods lacks standardisation. Most methods are tested on models of divergent size and connectivity in different tests, making it challenging to directly compare methods and exacerbated by the diversity of algorithm inputs. While reconstruction benchmarks are common, they are purely technical and are no indication of the biological relevance of inferred models, instead mostly being useful to assess tools’ resilience to noise in data and topology. Notably, only CellNetOptimizer is validated on unseen experimental data generated after the initial Boolean network inference effort in the original publication.

Of methods covered in this review, CellNetOptimizer [121], Griffin [122, 123], BoNesis [124], and RE:IN [125] are supported by follow-up publications detailing how Boolean networks inferred by these algorithms were experimentally validated, though it should be noted that these follow-ups largely stem from the authors behind the respective inference method itself.

A large-scale comprehensive benchmark covering the breadth of available inference approaches is needed for structured evaluation of different tools’ capabilities. Insights from Pušnik et al. and Jiang et al. suggest that most metrics are unable to conclusively contrast different approaches especially given different method inputs. Here, we propose key elements of a conceptual framework for future application in a large-scale benchmark:

1. **I**  **Selection of a common ground truth system**

For all covered methods, the biological context and input information for the benchmarks only sparsely overlap between methods (Table S1). We propose the generation of a canonical benchmark case covering all possible method inputs:

1. a prior knowledge network with per-edge confidence labels (definitive/putative)
2. a draft Boolean network model with update function properties (essential/monotonic)
3. continuous, time-resolved experimental data together with a binarised version
4. a set of initial system states and expected steady states
5. a perturbation array with expected outcomes

Given interoperability issues between algorithms, each element of the canonical benchmark case requires conversion into the correct input format for technical consistency, which must be validated before any testing.

1. **II**  **A set of challenges for benchmarking**

Based on types of benchmarks shown in Table S1, we propose the following challenges for benchmarking:

1. Reconstruction benchmark – Generate synthetic data (with and without noise) from a reference Boolean model to be used together with a modified version of the reference model that introduces gaps and false positive and negative connections, aiming to infer the reference model via structural and dynamical similarity. This addresses technical robustness and resilience to noise.
2. Cross-validation benchmark – Infer Boolean networks using part of the experimental data, time series, perturbations, or expected behaviour and score prediction performance on unseen inputs. This addresses generalisability of inferred models for each method.
3. Experimental validation – Test how well predictions made by Boolean networks inferred from each method match experimental outcomes performed after inference. This is the most challenging benchmark considering the cost and time required for perturbation experiments, but stands as the only option for biological validation of inference quality.

1. **III**  **Controlled confounders**

To avoid variability stemming from external factors, we suggest the following ways to control common confounders:

1. Updating scheme – Point attractors are independent of updating scheme and provide an even metric to compare all methods. Methods employing the asynchronous updating scheme may be compared amongst each other.
2. Discretisation – All applicable methods should be evaluated on binary data discretised using the same method. Despite our considerations of k-means clustering compared to e.g. multi-scale binarisation [96], LogicGep and MIBNI internally force k-means clustering. To avoid a confounding effect stemming from different discretisation methods, we nonetheless recommend using k-means clustering.
3. Scale and connectivity – Test for scalability of inference methods; based on the review by Kadelka et al. [126], we suggest networks with 15, 30, and 50 nodes and maximum in-degree 6. For technical benchmarking, inferring very large models with 100, 200, and 300 nodes can also be insightful.
4. Replicability – Heuristic methods perform stochastic explorations of the search space and can infer different models across several runs. As such, outcomes for heuristic methods should be considered in the context of a distribution generated over multiple runs.

1. **IV**  **Comparative reporting**

Considering the variety of algorithm inputs and optimisation strategies, comparison metrics should be adapted to correctly frame each method within its capabilities:

1. Capability matrix – Declare which inputs each method uses and which dynamic features it can express. Conditions beyond the capability of a given method are marked as N/A and are not penalised.
2. Input informativity – For a comparison of purely data-driven algorithms versus algorithms supported by prior knowledge, assess performance of prior knowledge-assisted methods with decreasingly informative prior knowledge networks (e.g. scrambling or removing interactions)
3. Standardised output – A method-condition-metric matrix allows for conclusions like ‘method X performs best in regime Y for goal Z’, enabling comparison between similar methods.

The above framework for benchmarking Boolean network inference aids in reducing confounding effects and emphasises testing on comparable input information. Executing such a benchmark requires considerable effort in converting various input formats and extracting reporting metrics from each method, but is crucial in comparing different approaches. Due to the diversity of method capabilities, metrics should always be interpreted with respect to each algorithm’s implementations rather than relying purely on numeric interpretation.

## Conclusions

When selecting a tool for Boolean network inference, considerations should go beyond the optimisation algorithm itself, given that each method has important strengths and caveats that must be considered.

### Handling ambiguity in prior knowledge

Most approaches discussed in this review utilise prior knowledge on biomolecule interactions to guide the optimisation process, significantly restricting the search space and thus allowing inference of larger models. As noted earlier (see ‘Automatic and Manual Network Curation’), such knowledge can be compiled by biocurators using biological expertise or be derived using automated, data-driven approaches, prompting important considerations on prior knowledge quality. Several inference methods, including CANTATA, Sketchbook, Griffin, and RE:IN, allow the explicit specification of ambiguous or uncertain interactions.

As such, these methods can benefit from liberal extension of prior knowledge networks with putative interactions before pruning erroneous relationships during inference. Other inference methods, e.g. BoNesis, may be used in a similar way despite lacking granular encoding of confidence in prior knowledge. It should be noted that in CANTATA, unoptimised versions of the initial Boolean model are injected at each generation of the genetic program, necessitating particular attention to prior knowledge quality.

### Choice of update scheme

All analysed inference methods besides BoNesis, Sketchbook, and RE:IN utilise the synchronous updating scheme to determine dynamics accuracy between model predictions and experimental data and other constraints, though both synchronous and asynchronous updating schemes may be unable to accurately identify all plausible attractors for a given system.

For instance, attractors can be artefacts of the synchronous updating scheme rather than biologically meaningful states [127, 128]. These false positive steady states contribute to overestimation of inference accuracy when biologically meaningless attractors inflate the likelihood of being matched to experimental data. Conversely, synchronous attractor search can also produce false negatives given the rigour of deterministic updates.

The asynchronous updating scheme may be regarded as an alternative approximation of biological dynamics [129, 130], though its stochastic nature implies that true attractors of the system may not be identified because they are never sampled when traversing the search space.

Moreover, CellNetOptizimer and caspo employ a signal propagation scheme that may not account for complex, yet common topological features like feedback loops. BoNesis addresses failures to identify plausible attractors by implementing the most permissive Boolean network semantics, able to reproduce all behaviours of quantitative models at the cost of increased false positives [88, 108].

### Scalability considerations

Most permissive Boolean network semantics enable BoNesis to infer large models with hundreds of nodes. Similarly, other modern methods like Sketchbook and Griffin implement efficient computational representations and steady state specifications to infer models of considerable size. MIBNI and ATEN can efficiently infer BFs with many regulators, though the maximum number of regulators must be user-set; generally, prior-knowledge guided inference methods enable inference of larger models compared to data-only methods. Interestingly, a recent meta-analysis [126] of 122 distinct, expert-curated Boolean network models found the mean and median model sizes to be 42 and 23 nodes, respectively, with the biggest included model reaching 302 nodes. Average connectivity did not exceed 6.5. These insights based on manually curated models suggest that there may be an upper bound to the utility of inferring very large or highly connected models—the review by Pušnik et al. [32] suggests that algorithms like MIBNI overestimate connectivity, resulting in structurally implausible models.

In addition, analysing and interpreting the results of very large Boolean network models is challenging given that the states of many different nodes need to be considered for a biological conclusion, further restricted by increasing cost and time of experimental validation. Conversely, the modest mean and median model scales discussed by Kadelka et al. could also be indicative of a cognitive limit in constructing and analysing large models [131], rather than reflecting biological relevance of moderately-sized models. This cognitive limit could be addressed by automatically inferring Boolean network models using the methods covered in this review.

Interestingly, all approaches besides LogicGep check inference accuracy on entire candidate models, though methods like MIBNI and ATEN maximise mutual information and clause importance for individual BFs before combining them into a network model for evaluation. Methods beyond the scope of this review, like MIFuGP [82] optimise individual BFs independently before ultimately combining them into a network model. A similar idea is implemented in TaBooN [132], which implements a two-stage optimisation procedure, first independently inferring individual BFs before combining them into a globally optimal network. The approach benefits from parallelisation but may suffer in accuracy since it is ignorant of how functions influence each other [133, 134].

### Data discretisation challenges

The binary nature of biomolecule states in Boolean models imposes a significant loss of information during discretisation. The strict, exact nature of RE:IN, Sketchbook, caspo, Griffin, and BoNesis implies that inherent biological noise and information loss from a discretisation procedure could prevent acceptance of valid Boolean models. Additionally, the impact of discretisation is demonstrated by the finding that different discretisation procedures may result in a different set of inferred Boolean models [31].

### Boolean network inference for biological insights

Beyond producing context-specific and data-informed Boolean models automatically, the inference process can produce novel biological insights. Both heuristic and exact optimisation approaches invariably identify multiple models as optimal or consistent, reflecting limitations in both prior knowledge and noisy experimental measurements, and degeneracy in biological systems [81]. Indeed, the same outcome may be achieved using a range of processes [135], suggesting that Boolean network inference algorithms are directly applicable to gain biological knowledge by identifying which molecular mechanisms can account for the same dynamics. Testing whether inferred models are able to successfully produce expected system dynamics beyond those used during the inference process can indicate how well these models generalise and reinforce novel biological insights, demonstrated for e.g. CANTATA [81].

Methods like caspo, BoNesis, and RE:IN provide analysis of inferred models to delineate e.g. critical and mutually exclusive interactions, emphasising their roles in reproducing experimental observations. LogicGep makes use of continuous experimental data to resolve ties between inferred BFs, addressing degeneracy in inferred models.

Structural analysis of sets of inferred models can reveal common motifs leading to the same dynamic behaviour, highlighting them for experimental validation. The set of optimal models produced by any inference method is informative for determining interaction relevance through post hoc analysis. For instance, perturbation and sensitivity analyses highlight how specific interactions contribute to overall model dynamics and can be used to refine the set of inferred Boolean models for downstream analysis.

### Summary

Ultimately, the selection of an inference method depends on the type and extent of available experimental data, prior knowledge, or hypotheses of the system to be modelled. This review is not exhaustive, and there are numerous interesting inference approaches not discussed here, typically because they are not publicly available or rely on commercial software for inference; e.g. MIFuGP implements mutual information together with a fuzzy logic control strategy to control BF size [82], TaBooN uses both local and global optimisation procedures with Tabu search [132].

As mentioned in ‘Evaluation and Benchmarking’, a comprehensive, large-scale benchmarking effort would provide objective comparisons between methods which we aim to perform in the future according to the conceptual framework laid out in this work, complementing our review.

**Key Points**

- Method selection should be influenced by available data and prior knowledge: the choice between heuristic and exact inference methods depends on the type and extent of prior knowledge, experimental data, and modelling objectives.
- Prior knowledge is a key influence of inference outcomes: methods that explicitly encode uncertainty in interactions (CANTATA, Sketchbook, Griffin, RE:IN) can leverage liberal extension of prior knowledge networks, while methods lacking this granularity require greater attention to input quality.
- Data discretisation is a considerable challenge in Boolean network inference, shaping the evaluation of dynamic properties during the inference process.
- Systematic, inter-method benchmarking is lacking, preventing meaningful comparisons across approaches.
- Boolean network inference is a powerful tool to integrate prior knowledge with experimental data, and may be useful beyond automatic generation of Boolean network models to reveal novel biological mechanisms and degeneracy.

## Supplementary Material

Supplementary_Information_bbag499(1)

Table_S1_bbag499

1_symbolic_tree_bbag499

2_hypergraph_bbag499

3_binary_decision_diagram_bbag499

4_bit_vector_bbag499

5_k_expression_bbag499

## Data Availability

No data was generated or analysed in the writing of this article. This review is based on manual literature synthesis of previously published scientific literature.
