# Supplementary material for: From prior knowledge to data-informed models: a review of Boolean network inference
Source: Brief Bioinform. 2026 Sep 15;27(5):bbag499. doi: 10.1093/bib/bbag499 (PMC13577669; doi:10.1093/bib/bbag499)
Supplement: Supplementary file 1 [file supplementary_information_bbag499(1).docx]

Supplementary Information

**S1 - Rationale for included methods and detailed Europe PMC query**

We queried Europe PMC for Boolean model inference and chose to review publicly available Boolean network inference tools covering heuristic and exact optimisation approaches to demonstrate trade-offs between inference scalability, accuracy, and optimality. We included both purely data-driven methods (e.g. MIBNI [83]) and approaches using prior knowledge with experimental data (e.g. RE:IN [94]), highlighting complementary aspects of either approach and use cases. While most approaches reason under the synchronous updating scheme with basic Boolean functions (e.g. CANTATA [81]), we also introduce methods capable of producing Boolean network models under asynchronous updating schemes with complex Boolean functions (e.g. Sketchbook [92]). The reviewed approaches range from older, yet fundamental works (e.g. CellNetOptimizer [86]), to novel methods under active testing and development (e.g. Sketchbook [92]).

We queried Europe PMC using the following keywords: *("Boolean network" OR "logical model" OR "logic-based model" OR "logic model") AND (inference OR reconstruction OR "network inference" OR "model inference" OR learning OR optimisation OR calibration OR reasoning OR identification),* and performed this query for the last time on 16/12/2025.
