# Supplementary material for: From prior knowledge to data-informed models: a review of Boolean network inference
Source: Brief Bioinform. 2026 Sep 15;27(5):bbag499. doi: 10.1093/bib/bbag499 (PMC13577669; doi:10.1093/bib/bbag499)
Supplement: Supplementary file 2 [file table_s1_bbag499.docx]

*Table S1 - Benchmarks performed for each method in the original publications together with the tested biological scenario and data type used for the benchmark (N/A = not applicable).*

*Alt text: A table with the following headers: Method, benchmark type, biological scenario, data type. The table shows, for each method covered in this review, how methods were tested or benchmarked in the original publication along with references to the prior knowledge network/model used for testing to add biological context. The “data type” column elaborates on whether methods used experimental or purely synthetic data or dynamic constraints.*

| **Method** | **Benchmark type** | **Benchmark description** | **Biological scenario** | **Data type** |
| --- | --- | --- | --- | --- |
| CANTATA | Reconstruction | Infer original BN from data and scrambled version of original BN | Fission yeast cell cycle [1] | Synthetic |
|  | Cross-validation | Assess how well inferred BNs generalise to unseen perturbation data | Hepatocellular carcinoma, DREAM4 challenge [2] | Experimental |
|  | Gap prediction | Infer missing interactions such that the BN matches a set of supposed attractors | Arabidopsis thaliana stem cells [3] | Experimental |
| CellNetOptimizer | Reconstruction | Infer original BN from data and prior knowledge network | EGFR/TNFR signaling | Synthetic |
|  | Reconstruction | Infer reference BN from experimental data and scrambled versions of reference BN | Hepatocellular carcinoma | Experimental |
|  | Reconstruction | Infer reference BN from experimental data and randomised signaling networks with similar topology as reference BN |  |  |
|  | Cross-validation | Assess how well inferred BNs generalise to unseen perturbation data |  |  |
|  | Cross-validation | Assess how well inferred BNs generalise to novel experimental perturbation data |  |  |
|  | Noise resilience | Assess impact of scrambling experimental data on inference performance |  |  |
|  | Model extension | Evaluate how new edges improve model’s fit to data |  |  |
| caspo | Reconstruction | Infer original BN from data with Gaussian noise and prior knowledge network | Hepatocellular carcinoma | Synthetic |
|  | Scalability | Assess how multi-value discretisation impacts inference outcomes |  |  |
|  | Consistency check | Determine whether the method identifies all optimal models |  |  |
| Sketchbook | Consistency check | Determine whether there is a model consistent with a prior knowledge network and binarised data | T cell survival in leukaemia [4] | Experimental |
|  | Gap prediction | Infer missing interactions such that the BN matches a set of supposed attractors | Arabidopsis thaliana sepal primordium polarity [5] | Experimental |
|  | Reconstruction | Infer original BN from a partially unspecified version of the original BN, its influence graph, update function properties, and dynamic properties | E protein, NSP4, electron transport chain, Interferon I, and NSP9 in COVID-19 [6], Macrophage activation [7] | Synthetic |
| BoNesis | Reconstruction | Infer BNs from random, scale-free directed graphs and dynamic properties | N/A – fully synthetic, random graphs | Synthetic |
|  | Consistency check | Determine whether there is a model consistent with a prior knowledge network and expected reachability between model states | Central nervous system development [8] | Synthetic |
| MIBNI | Reconstruction | Infer original BN from time-resolved data generated from original BN | N/A – fully synthetic, random graphs | Synthetic |
|  | Reconstruction | Infer original BN from time-resolved data generated from original BN | E. coli [9] | Synthetic |
|  | Reconstruction | Infer BN from gene expression dataset | E. coli, DREAM3 challenge [2] | Experimental |
|  | Reconstruction | Infer BN from gene expression dataset | Fission yeast cell cycle [1] | Experimental |
| ATEN | Reconstruction | Infer original BN from (noisy) time-resolved data generated from original BN | N/A – fully synthetic BNs | Synthetic |
|  | Reconstruction | Infer target BN from (noisy) time-series data relating to target BN | Drosophila segment polarity [10,11] | Experimental |
| RE:IN | Consistency check | Determine whether there is consistent BN and expected reachability between model states | Budding yeast cell cycle [12] | Experimental |
|  | Gap prediction | Infer missing interactions such that the BN matches a set of supposed attractors | Myeloid progenitor differentiation [13] | Experimental |
|  | Consistency check | Determine whether there is consistent BN and expected reachability between model states | Cardiac development in murine embryos [14] | Experimental |
| Griffin | Reconstruction | Infer a consistent BN given a regulation graph, and expected steady states | Arabidopsis thaliana floral organ determination [15] | Experimental |
|  | Gap prediction | Infer missing interactions such that the BN matches a set of supposed attractors | Arabidopsis thaliana sepal primordium polarity [5] |  |
|  | Consistency check | Determine whether there is consistent BN and expected reachability between model states | Arabidopsis thaliana root stem cell niche [16] |  |
|  | Scalability | Assess computational performance of clause learning versus naive encoding |  |  |
| LogicGep | Reconstruction | Infer original BN from (noisy) time-resolved data generated from original BN | N/A – fully synthetic BNs | Synthetic |
|  |  |  | Murine hematopoietic stem cells [17] | Experimental |
|  |  |  | Murine embryonic stem cells [18] |  |

**References**

1. Davidich MI, Bornholdt S. Boolean Network Model Predicts Cell Cycle Sequence of Fission Yeast. *PLOS ONE* 2008;**3**(2):e1672. https://doi.org/10.1371/journal.pone.0001672.

2. Prill RJ, Marbach D, Saez-Rodriguez J *et al.* Towards a Rigorous Assessment of Systems Biology Models: The DREAM3 Challenges. *PLOS ONE* 2010;**5**(2):e9202. https://doi.org/10.1371/journal.pone.0009202.

3. Azpeitia E, Benítez M, Vega I *et al.* Single-cell and coupled GRN models of cell patterning in the Arabidopsis thaliana root stem cell niche. *BMC Syst Biol* 2010;**4**(1):134. https://doi.org/10.1186/1752-0509-4-134.

4. Saadatpour A, Wang RS, Liao A *et al.* Dynamical and Structural Analysis of a T Cell Survival Network Identifies Novel Candidate Therapeutic Targets for Large Granular Lymphocyte Leukemia. *PLOS Comput Biol* 2011;**7**(11):e1002267. https://doi.org/10.1371/journal.pcbi.1002267.

5. La Rota C, Chopard J, Das P *et al.* A data-driven integrative model of sepal primordium polarity in Arabidopsis. *Plant Cell* 2011;**23**(12):4318–33. https://doi.org/10.1105/tpc.111.092619.

6. Ostaszewski M, Mazein A, Gillespie ME *et al.* COVID-19 Disease Map, building a computational repository of SARS-CoV-2 virus-host interaction mechanisms. *Sci Data* 2020;**7**(1):136. https://doi.org/10.1038/s41597-020-0477-8.

7. Raza S, Robertson KA, Lacaze PA *et al.* A logic-based diagram of signalling pathways central to macrophage activation. *BMC Syst Biol* 2008;**2**(1):36. https://doi.org/10.1186/1752-0509-2-36.

8. Qiu X, Mao Q, Tang Y *et al.* Reversed graph embedding resolves complex single-cell trajectories. *Nat Methods* 2017;**14**(10):979–82. https://doi.org/10.1038/nmeth.4402.

9. Gama-Castro S, Salgado H, Peralta-Gil M *et al.* RegulonDB version 7.0: transcriptional regulation of Escherichia coli K-12 integrated within genetic sensory response units (Gensor Units). *Nucleic Acids Res* 2011;**39**(Database issue):D98-105. https://doi.org/10.1093/nar/gkq1110.

10. Albert R, Othmer HG. The topology of the regulatory interactions predicts the expression pattern of the segment polarity genes in Drosophila melanogaster. *J Theor Biol* 2003;**223**(1):1–18. https://doi.org/10.1016/s0022-5193(03)00035-3.

11. Vera-Licona P, Jarrah A, Garcia-Puente LD *et al.* An algebra-based method for inferring gene regulatory networks. *BMC Syst Biol* 2014;**8**:37. https://doi.org/10.1186/1752-0509-8-37.

12. Li F, Long T, Lu Y *et al.* The yeast cell-cycle network is robustly designed. *Proc Natl Acad Sci* 2004;**101**(14):4781–6. https://doi.org/10.1073/pnas.0305937101.

13. Krumsiek J, Marr C, Schroeder T *et al.* Hierarchical Differentiation of Myeloid Progenitors Is Encoded in the Transcription Factor Network. *PLOS ONE* 2011;**6**(8):e22649. https://doi.org/10.1371/journal.pone.0022649.

14. Herrmann F, Groß A, Zhou D *et al.* A Boolean Model of the Cardiac Gene Regulatory Network Determining First and Second Heart Field Identity. *PLOS ONE* 2012;**7**(10):e46798. https://doi.org/10.1371/journal.pone.0046798.

15. Alvarez-Buylla ER, Benítez M, Corvera-Poiré A *et al.* Flower development. *Arab Book* 2010;**8**:e0127. https://doi.org/10.1199/tab.0127.

16. Azpeitia E, Weinstein N, Benítez M *et al.* Finding Missing Interactions of the Arabidopsis thaliana Root Stem Cell Niche Gene Regulatory Network. *Front Plant Sci* 2013;**4**. https://doi.org/10.3389/fpls.2013.00110.

17. Hamey FK, Nestorowa S, Kinston SJ *et al.* Reconstructing blood stem cell regulatory network models from single-cell molecular profiles. *Proc Natl Acad Sci* 2017;**114**(23):5822–9. https://doi.org/10.1073/pnas.1610609114.

18. Guo G, Huss M, Tong GQ *et al.* Resolution of cell fate decisions revealed by single-cell gene expression analysis from zygote to blastocyst. *Dev Cell* 2010;**18**(4):675–85. https://doi.org/10.1016/j.devcel.2010.02.012.
